# The impact of vaping on periodontitis: A systematic review

**DOI:** 10.1002/cre2.360

**Published:** 2020-12-04

**Authors:** Carlos Alberto Figueredo, Nancy Abdelhay, Carlos Marcelo Figueredo, Raisa Catunda, Monica Prasad Gibson

**Affiliations:** ^1^ Faculty of Medicine and Dentistry University of Alberta Edmonton Canada; ^2^ School of Dentistry and Oral Health Griffith University Gold Coast Australia; ^3^ Faculty of Dentistry Alexandria University Egypt

**Keywords:** e‐cigarettes, periodontitis, review, vaping

## Abstract

**Background and objective:**

While tobacco cigarette smoking has been proven to be a risk factor for periodontitis, limited information is available regarding vaping, a new alternative to smoking that has been branded as less harmful. Several important in vitro studies have shown that vaping has a similarly damaging effect as cigarette smoking on the health of the periodontium. However, a comprehensive review is lacking in this field. Therefore, we aimed to systematically review the literature about the impact of vaping on periodontitis.

**Methods:**

The research question was created using the PICOs format. A systematic search of the following electronic databases was performed up to March 2020: Medline, Embase, PubMed, Cochrane, and grey literature. Human studies that assessed periodontal status (plaque index, bleeding on probing, clinical attachment loss, marginal bone loss, and probing depth) in e‐cigarette users compared to non‐smokers (control group) were assessed based on an estimate of fixed effects. The weights of the studies were calculated based on their risks of bias.

**Results:**

After duplicates were removed, 1,659 studies were screened and 8 case–control studies that investigated the relationship between vaping and periodontal parameters in humans were selected after their risk of bias assessment. Estimated effects of vaping after weighting results based on their standard deviation showed increased plaque, marginal bone loss, clinical attachment loss, pocket depth, and reduced bleeding on probing.

**Conclusion:**

This study concluded that there is not enough evidence to fully characterize the impacts of vaping on periodontitis. However, within the limitations of our review and the selected included studies, the available results point to increased destruction of the periodontium leading to the development of the disease.

## 1 INTRODUCTION

Periodontal diseases are defined as an inflammatory process associated with bacterial activity and mediated by the host's immunologic response (Armitage, 1999; Tonetti et al., 2018). This aggression may result in the loss of connective tissue attachment and consequently bone loss (Armitage, 1999; Tonetti et al., 2018). Gingivitis, a reversible form of periodontal disease, is initially characterized by gingival inflammation caused by bacterial colonization forming the biofilm (Armitage, 1999; Tonetti et al., 2018). In susceptible individuals, gingivitis may progress to an irreversible form of the disease, periodontitis, where there is the loss of periodontal ligament and apical migration of the junctional epithelium (Armitage, 1999; Tonetti et al., 2018). Risk factors, such as diabetes, genetics, and smoking, have been related to the susceptibility, prevalence, and severity of the disease (Armitage, 1999; Tonetti et al., 2018).

It is well established that cigarette smoking is considered as a risk factor in the development of periodontitis (Armitage, 1999; Leite et al., 2018; Tonetti et al., 2018). It has been shown that patients who smoke suffer from more severe forms of periodontitis (Javed et al., 2013). Disease progression is directly related to the frequency of smoking, where heavy smokers show more severe forms of the disease compared to light smokers (Tonetti et al., 2018). Different studies categorize the frequency of smoking differently but according to one review, smoking less than 9 cigarettes per day is considered light, and more than 31 is considered heavy smoking (Johnson & Guthmiller, 2000). A recent systematic review with meta‐analysis showed that smoking increases the risk of developing periodontitis by 85% (Leite et al., 2018). Smoking also impacts the response to periodontal treatment; smokers show only 50%–75% improvement in their clinical parameters after scaling and root planing compared to non‐smokers (Tonetti et al., 2018). Another study analyzed the effects of cigarette smoking on periodontal parameters and found significant increases in plaque index, pocket depth, and clinical attachment loss levels in cigarette smokers compared to non‐smokers (Javed et al., 2017). It has been evidenced that tobacco smoking results in a proinflammatory effect by stimulating the secretion of specific cytokines and radical oxygen species (ROS) that play a role in the destruction of periodontal tissues (Katz et al., 2005).

Vaping electronic cigarettes (e‐cigarettes) have grown into a popular recreational activity among teenagers and young adults in Canada in the last few years. Since the Tobacco and Vaping Products Act (TVPA) became legal in Canada in May 2018, adults have the right to purchase vaping products with nicotine as an allegedly less harmful option than smoking (Government of Canada: Smoking, Vaping, and Tobacco, 2020). Instead of burning tobacco, as traditional cigarettes do, e‐cigarettes heat up and vaporize nicotine or other flavoring products that might be included in it. The Canadian Tobacco, Alcohol and Drugs Survey conducted on students between grades 7 and 12 between 2016 and 2017 concluded that 23% of students in grades 7–12 had tried a vaping product at least once. Ten percent reported using them within the last 30 days and 53% of all students thought it would be” fairly easy” or” very easy” to get a vaping product if they wanted one (Government of Canada: Smoking, Vaping, and Tobacco, 2020). Interestingly, while 74% of current smokers recognize a great risk in smoking traditional cigarettes regularly, only 24% of current vape users recognize risk in vaping electronic cigarettes.

The literature on vaping e‐cigarettes has shown that there are systemic impacts of vaping (Gaur & Agnihotri, 2019; Government of Canada: Smoking and Oral Cancer, 2011). Vaping with nicotine exposes users to nicotine addiction and side effects such as altered teen brain development and cognitive and behavioral problems (Government of Canada: Smoking and Oral Cancer, 2011). Vaping without nicotine still proposes risks of exposure to the chemicals that are released in the heating process of the device, such as aluminum, copper, and lead (Gaur & Agnihotri, 2019). E‐cigarettes also pose a hazard for traumatic injuries. Blast injuries caused by battery explosion are also an associated risk, mainly in countries where there is no regulation on the manufacture and safety of e‐cigarettes (Kite et al., 2016). An in vitro study correlated e‐cigarette aerosol exposure to DNA damage and mitochondrial dysfunction in lung fibroblasts (Lerner et al., 2016). Another study compared the effects of cigarette smoke and e‐cigarette aerosol in bone marrow‐derived mesenchymal stem cells and found that e‐cigarette aerosol exposure causes overproduction of ROS (Shaito et al., 2017). Focusing on a correlation between oral health and vaping, a recent study showed that e‐cigarette exposure‐mediated carbonyl stress leads to increased levels of prostaglandin‐E2 and cyclooxygenase‐2 in human gingival epithelium compared to control (Lerner et al., 2015). Several studies, such as the ones included in this review, analyzed the impact of vaping on periodontal parameters and found increased levels of plaque index, pocket depth, clinical attachment loss, and marginal bone loss in vaping groups compared to non‐smokers (Al‐Aali et al., 2018; AlQahtani et al., 2018, 2019; ArRejaie et al., 2019; BinShabaiba et al., 2019; Javed et al., 2017; Mokeem et al., 2018; Vohra et al., 2020). Despite all the evidence that smoking can negatively affect the periodontal tissues, there is still little evidence about the impact of vaping. Based on the available literature, we hypothesize that we may find similar clinical periodontal manifestations in vapers as seen in cigarette smokers. Therefore, we aimed to systematically review available evidence about the impact of vaping e‐cigarettes on periodontal statuses.

## 2 MATERIALS AND METHODS

### 2.1 Protocol, registration, conduct, and reporting

This systematic review was conducted according to the Cochrane Handbook (Cochrane handbook for systematic reviews of interventions, 2020), and adhered to the Preferred Reporting Items for Systematic Review and Meta‐Analyses (PRISMA) to ensure the higher methodological quality of the study. The protocol for this systematic review was registered in Prospero – International prospective register of systematic reviews (Centre for reviews and dissemination, University of York, York, United Kingdom) under ID CRD42018114837 (International Prospective Register of Systematic Reviews, PROSPERO, 2011). The authors have stated explicitly that there are no conflicts of interest in connection with this article.

### 2.2 Eligibility criteria

This systematic review only included human studies that investigated an association between vaping and periodontal status in a clinical context. Studies that included parameters such as plaque index (PI), bleeding on probing (BOP), clinical attachment loss (CAL), probing depth (PD), and marginal bone loss (MBL) were included. Also, these studies included patients who did not receive periodontal treatment 6 months before the study. No age or sex restrictions were applied.

### 2.3 Exclusion criteria

Descriptive studies, case reports, case series, abstracts, systematic/scope reviews, and expert opinions were not included. Exclusion criteria for patients: pregnancy, current cigarette smokers, waterpipe smokers, or smokeless tobacco users, immunocompromised patients, diabetic patients, patients who went through periodontal therapy during the last 6 months, patients taking anti‐inflammatory or antibiotic medication, and edentulous patients.

### 2.4 PICO

The researchable question was created using the PICOs (Population, Intervention, Comparison, Outcome) format. Population: Group A inclusion criteria: Participants who are not using any form of tobacco; ages 13 and up. Group B inclusion criteria: Participants who reported vaping e‐cigarettes for at least 1 year before the study. Intervention: History of vaping of e‐cigarettes. Comparison: Non‐smokers. Outcomes: Presence of periodontal disease as indicated by increased probing depth, attachment loss, gingival recession, and bone resorption.

### 2.5 Search strategy

A systematic search of the following electronic databases was performed up to March 2020: Medline, Embase, PubMed, and Cochrane. Additional information about vaping laws and prevalence statistics were obtained from the Canadian government website. The search was restricted to the English language and no year restriction was applied. The search strategy used tried to include all terminology that refers to electronic cigarettes and periodontal parameters.

### 2.6 Study selection

A two‐phase process to select the final articles was followed. In the first phase, two reviewers (CF and RC) independently screened titles and abstracts from all gathered references. In the second phase, full‐text articles were assessed by reviewers to confirm their final selection. In the case of disagreements, the consensus was reached after discussion, a third person (NA) was involved when necessary.

### 2.7 Risk of bias in individual studies

The studies were evaluated by 2 reviewers following The Joanna Briggs Institute (JBI) critical appraisal checklist tool (Joanna Briggs Institute critical appraisal tools, 2017). The critical appraisal checklist tool for analytical cross‐sectional studies was used to assess the risk of bias. This tool of assessment describes the following main components: true random sequence generation, allocation concealment, blinded outcome assessment, selective outcome assessment, and appropriate statistical analysis. Studies would be assigned to the highest risk of bias if the sequence generation and allocation concealment were unclear.

### 2.8 Data synthesis

SPSS was used to make a linear mixed model of the extracted results. In this model, the dependent variables are the mean values being affected by the factor of vaping or not. Mean values are weighted by their standard deviations (weight = 1/SD). The linear mixed model provides an estimate of fixed effects that vaping is causing on each clinical measurement. Each parameter has a value representing the difference between vaping and control groups.

## 3 RESULTS

### 3.1 Studies selection

**FIGURE 1:**
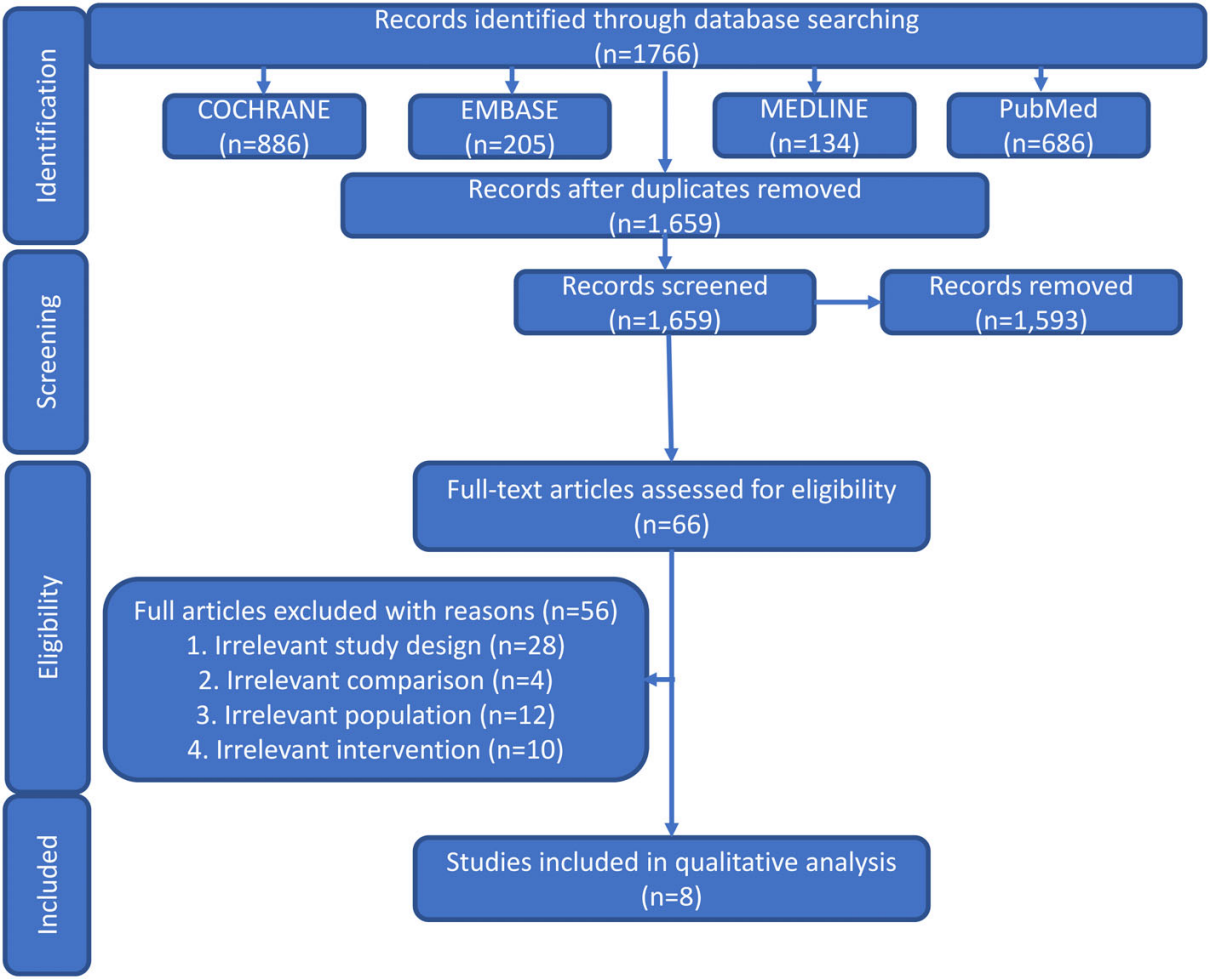
Flow diagram of literature search divided into the identification, screening, eligibility, and phases. Reasons for study exclusions are included in the eligibility phase

The search found 1766 studies across 4 databases as demonstrated in the PRISMA flowchart (Figure 1). Duplicates were removed and 1659 studies were screened. After title and abstract reading, 66 studies were selected for full‐text reading. From these, 8 studies fit in our inclusion criteria and were suitable for fulfilling our research question. The studies that were not included due to full‐text assessment were excluded due to irrelevant study design, comparison, intervention, or population.

### 3.2 Study characteristics

Study characteristics are reported in Table 1. All eight studies (Al‐Aali et al., 2018; AlQahtani et al., 2018, 2019; ArRejaie et al., 2019; BinShabaiba et al., 2019; Javed et al., 2017; Mokeem et al., 2018; Vohra et al., 2020) had a cross‐sectional design and they were all carried out in Saudi Arabia. They had a combined sample size of 582 (574 males and 8 females) ranging from 24 to 45 years of age. All studies were published between 2017 and 2019. History of vaping habits ranged from 0.9 to 8.7 years.

**TABLE 1:** Study characteristics are divided into the year, country and journal of publication, study design, inclusion criteria for included groups, sample, and vaping habit characteristics

Author, year	Country	Published on	Study design	Vaping inclusion criteria	Non smoker inclusion criteria	Sample characteristics	Vaping habit characteristics
Javed et al. (2017)	Saudi Arabia	Journal of periodontology	Case‐control	Individuals without previous history of tobacco use who had been exclusively vaping e‐cigs for at least 12 months	Individuals who reported to have never used any form of tobacco product.	31 male e‐cig users with mean age of 37.6 ± 2.1 and 30 male never smokers with mean age of 40.7 ± 1.6.	6.8 ± 0.8 daily frequency and 2.2 ± 0.2 years of duration.
Mokeem et al. (2018)	Saudi Arabia	Environmental toxicology and pharmacology	Case‐control	Individuals without previous history of tobacco use who had been exclusively vaping e‐cigs for at least 12 months.	Individuals who reported to have never used any form of tobacco product.	38 male never smokers with mean age of 40.6 ± 4.5 and 37 male e‐cig users with mean age of 28.3 ± 3.5.	9.2 ± 1.4 daily frequency and 3.1 ± 0.4 years of duration.
Al‐Aali et al. (2018)	Saudi Arabia	Clinical implant dentistry and related research	Case‐control	Individuals who had been exclusively vaping for at least the past year.	Individuals who reported to have never used any form of tobacco product.	45 male never smokers with mean age of 42.6 ± 2.7 and 47 male e‐cig users with mean age of 35.8 ± 6.2.	6.5 ± 0.9 daily frequency and 4.4 ± 1.8 years of duration.
AlQahtani et al. (2018)	Saudi Arabia	Clinical implant dentistry and related research	Case‐control	Current e‐cig users	Individuals who reported to have never used any form of tobacco product.	40 male never smokers with mean age of 42.6 ± 2.7 and 40 male e‐cig users with mean age of 35.6 ± 7.1.	6.5 ± 0.9 daily frequency and 8.7 ± 3.8 years of duration.
BinShabaiba et al. (2019)	Saudi Arabia	Archives of Oral biology	Case‐control	Individuals exclusively using electronic‐cigarettes at least once daily were defined as “electronic‐cigarette users”	And individuals who reported to have never used any form of tobacco‐product were defined as “never smokers”	39 male and 6 female never smokers with mean age of 40.6 ± 3.3 and 42 male and 2 female e‐cig users with mean age of 36.5 ± 1.7	20.3 ± 3.5 daily minutes and 9.4 ± 2.6 years of duration.
Vohra et al. (2020)	Saudi Arabia	Journal of American college health	Case‐control	Self‐reported electronic cigarette users (individuals who were using electronic cigarette at least once daily as the sole source of nicotine intake)	Self‐reported never smokers	26 male never smokers with mean age of 33.5 ± 1.4 and 26 male e‐cig users with mean age of 31.6 ± 2.4.	30.2 ± 8.5 daily minutes and 0.9 ± 0.2 years of duration.
Alqahtani et al. (2019)	Saudi Arabia	Clinical implant dentistry and related research	Case‐control	Electronic‐cigarette users (individuals vaping once daily for at least 1 year	Nonsmokers (individuals who had never used tobacco in any form)	35 male never smokers with mean age of 32.2 ± 0.6 and 34 male e‐cig users with mean age of 33.5 ±0.7.	14.3 ± 1.2 daily frequency and 3.5 ± 0.6 years of duration.
ArRejaie et al. (2019)	Saudi Arabia	Journal of periodontology	Case‐control	Vapers who reported vaping e‐cigs for at least the past year	Participants who never consumed tobacco in any form during their lifetime	32 male never smokers with mean age 42.6 ± 2.7 and 32 e‐cig users with mean age of 35.8 ± 6.2	6.5 ± 0.9 daily frequency and 4.4 ± 1.8 years of duration.

*Note*: All studies were carried out in Saudi Arabia and had a case–control design.

### 3.3 Risk of bias within studies

The Joanna Briggs Institute assessment tool for risk of bias of cross‐sectional studies was used, results are reported in Table 2 (Joanna Briggs Institute critical appraisal tools, 2017). The risk of bias was categorized as high if the percentage of yes is equal or lower than 49, moderate if the percentage of yes was between 50% and 69%, and low if the percentage of yes was equal or higher than 70%. Following these criteria, four of the included studies were at low risk of bias and the other four at moderate risk of bias.

**TABLE 2:** Joanna Briggs Institute risk of bias tool for cross‐sectional studies

Author	Q1*	Q2*	Q3*	Q4*	Q5*	Q6*	Q7*	Q8*	%Yes
Javed et al. (2017)	Y	Y	N	Y	Y	Y	Y	Y	87
Mokeem et al. (2018)	Y	Y	N	Y	Y	Y	Y	Y	87
Al‐Aali et al. (2018)	Y	Y	N	Y	N	N	Y	Y	62
AlQahtani et al. (2018)	Y	Y	N	Y	N	N	Y	Y	62
BinShabaiba et al. (2019)	Y	Y	N	Y	Y	Y	Y	N	75
Vohra et al. (2020)	Y	Y	N	Y	N	N	Y	Y	62
Alqahtani et al. (2019)	Y	Y	N	Y	N	N	Y	Y	75
ArRejaie et al. (2019)	Y	Y	N	Y	N	N	Y	Y	62

Q1. Were the criteria for inclusion in the sample clearly defined?

Q2. Were the study subjects and the setting described in detail?

Q3. Was the exposure measured in a valid and reliable way?

Q4. Were objective, standard criteria used for measurement of the condition?

Q5. Were confounding factors identified?

Q6. Were strategies to deal with confounding factors stated?

Q7. Were the outcomes measured in a valid and reliable way?

Q8. Was appropriate statistical analysis used?

Q: Question.

Y: Yes. N: No.

* Y = yes, N = no.

### 3.4 Results of individual studies

**FIGURE 2:**
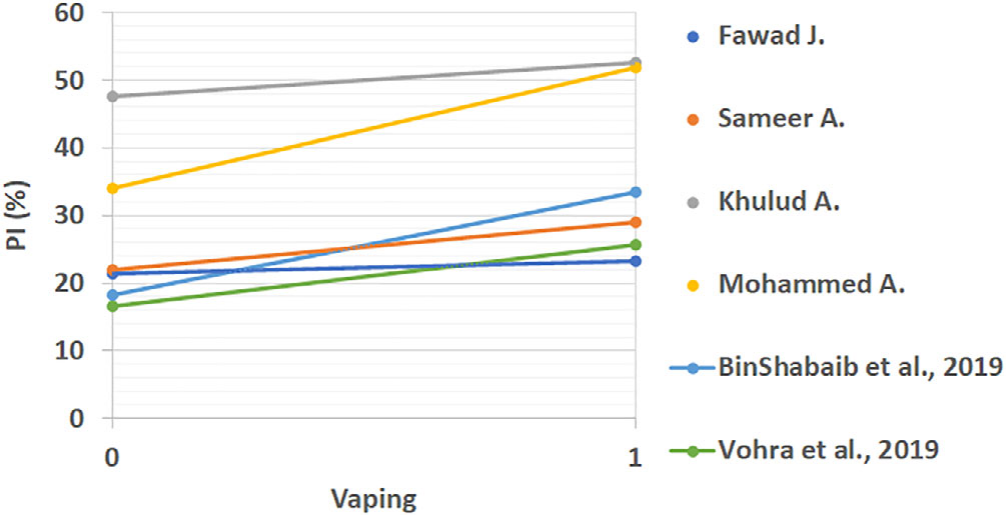
Linear plaque index (PI) comparison across studies (%). Where 0 is the control and 1 is the vaping group. PI results are consistently increased across studies with the use of e‐cigarettes

**FIGURE 3:**
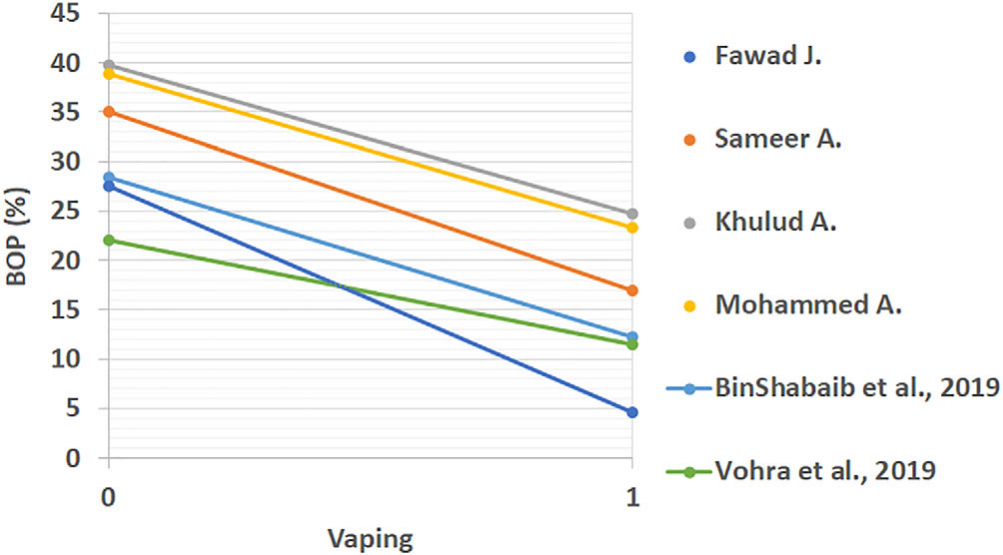
Linear bleeding on probing (BOP) comparison across studies (%). (%). 0 indicates the control group and 1 the vaping group. BOP results are consistently and dramatically lowered with e‐cigarette intervention

**FIGURE 4:**
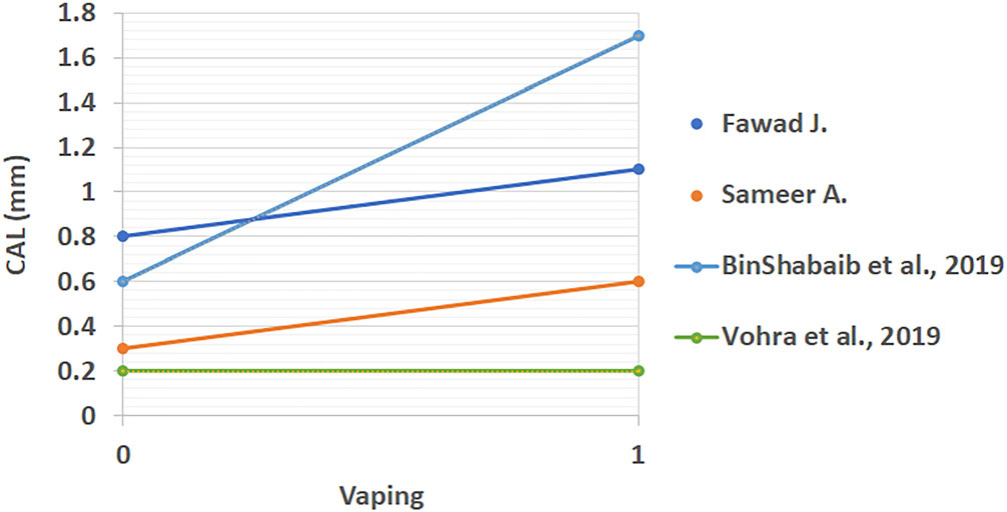
Linear clinical attachment loss (CAL) comparison across studies (mm) (%). 0 indicates the control group and 1 the vaping group. CAL results are increased with e‐cigarette intervention in all studies with exception to Vohra et al

**FIGURE 5:**
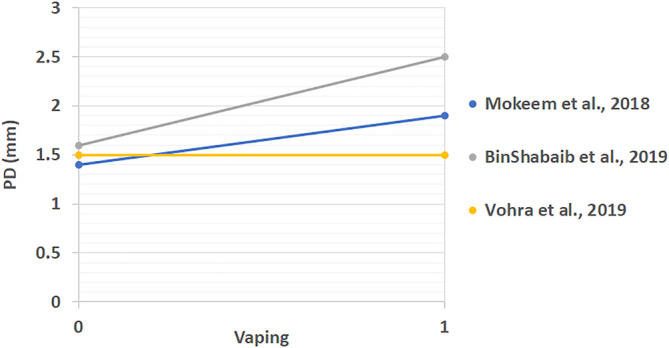
Linear probing depth (PD) comparison across studies (mm) (%). 0 indicates the control group and 1 the vaping group. PD results are increased with e‐cigarette intervention in all studies with exception to Vohra et al

**FIGURE 6:**
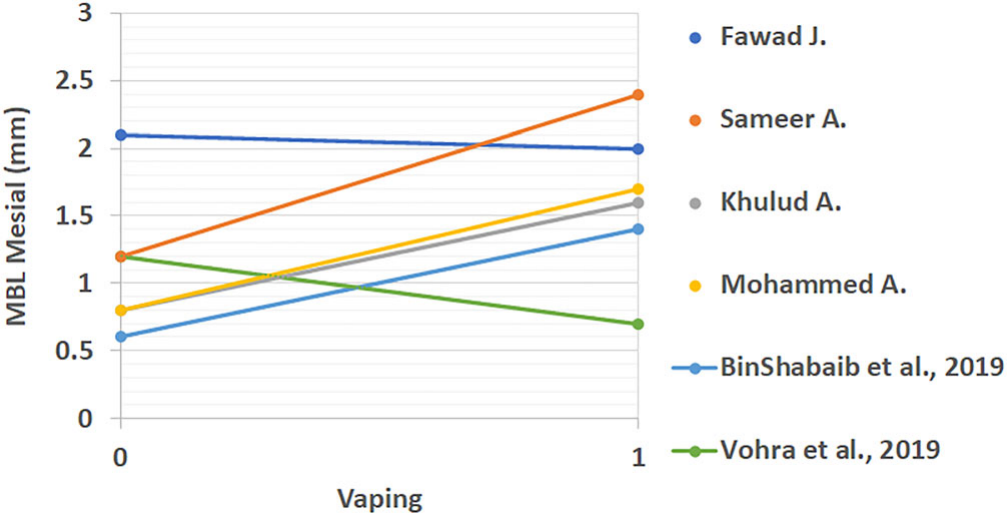
Linear mesial marginal bone loss comparison across studies (mm). (%). 0 indicates the control group and 1 the vaping group. Mesial MBL results showed a trend to increasing with e‐cigarette intervention, however, some studies reported lower levels in vaping groups

**FIGURE 7:**
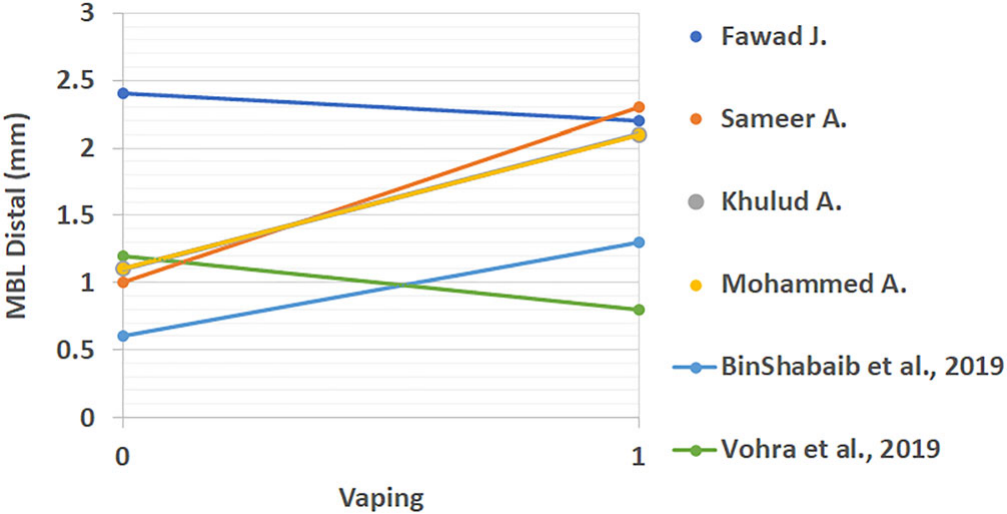
Linear distal marginal bone loss comparison across studies (mm) (%). 0 indicates the control group and 1 the vaping group. Distal MBL results showed a trend to increasing with e‐cigarette intervention, however, some studies reported lower levels in vaping groups. Figures (2, 3, 4, 5, 6, 7): Effect of vaping in mean values of periodontal parameters from each study, where control groups are represented by 0 and vaping groups are represented by 1. (2–3) Measures in percentage, and (4–7) measures in millimeters

All extracted results from included studies that compared the periodontal status of vape users and non‐smokers can be found in Table 3. These studies looked at differences between PI, BOP, CAL, PD, MBL mesial, and MBL distal. The linear comparison of these results is shown in Figures 2, 3, 4, 5, 6, 7. The results in Table 3 are divided into mean values and standard deviation of the means. From all the periodontal parameters, only BOP and PI were reported by all eight shortlisted studies. Mean values for BOP were consistently higher in control compared to vaping groups. Meanwhile, PI values were consistently higher in vaping groups compared to control.

**TABLE 3:** Extracted study results with means and standard deviations

Author	Vaping	PI (mean)	PI (SD)	BOP (mean)	BOP (SD)	CAL (mean)	CAL (SD)	PD mm (mean)	PD mm(SD)	MBL mesial (mean)	MBL mesial (SD)	MBL distal (mean)	MBL distal (SD)
Javed et al. (2017)	0	21.4	2.8	27.5	3.2	0.8	0.1	NA	NA	2.1	0.5	2.4	0.3
Javed et al. (2017)	1	23.3	3.4	4.6	2.9	1.1	0.2	NA	NA	2	0.6	2.2	0.4
Mokeem et al. (2018)	0	22	2	35	8	0.3	0.1	1.4	0.9	1.2	0.8	1	0.8
Mokeem et al. (2018)	1	29	3	17	3	0.6	0.2	1.9	0.9	2.4	0.7	2.3	0.7
Al‐Aali et al. (2018)	0	47.6	9.6	39.8	18.1	NA	NA	NA	NA	0.8	0.2	1.1	0.5
Al‐Aali et al. (2018)	1	52.6	11.9	24.7	5.3	NA	NA	NA	NA	1.6	0.7	2.1	1
AlQahtani et al. (2018)	0	34.1	14.7	38.9	19.6	NA	NA	NA	NA	0.8	0.2	1.1	0.5
AlQahtani et al. (2018)	1	51.9	10.2	23.3	5.1	NA	NA	NA	NA	1.7	0.6	2.1	1
BinShabaiba et al. (2019)	0	18.2	2.7	28.4	1.775	0.6	0.175	1.6	0.25	0.6	0.225	0.6	0.225
BinShabaiba et al. (2019)	1	33.4	2.525	12.2	1.525	1.7	0.225	2.5	0.3	1.3	0.35	1.4	0.325
Vohra et al. (2020)	0	16.6	2.1	22.1	3.3	0.2	0.02	1.5	0.2	1.2	0.3	1.2	0.2
Vohra et al. (2020)	1	25.6	6.2	11.5	0.8	0.2	0.04	1.5	0.3	0.8	0.06	0.7	0.05
Alqahtani et al. (2019)	0	12.6	1.1	19.8	1.3	NA	NA	0.8	0.1	NA	NA	NA	NA
Alqahtani et al. (2019)	1	27.2	2.4	6.6	1.3	NA	NA	3.2	0.3	NA	NA	NA	NA
ArRejaie et al. (2019)	0	29.7	5.2	39.3	18.1	NA	NA	NA	NA	0.8	0.2	1.1	0.5
ArRejaie et al. (2019)	1	43.5	8.1	14.7	5.3	NA	NA	NA	NA	1.2	0.7	1.6	1

*Note*: Percentage of plaque index (%). Percentage of bleeding on probing (%). Clinical attachment loss in millimeters (mm). Probing depth in millimeters (mm). Marginal bone loss in millimeters (mm).

Abbreviations: BOP, bleeding on probing; CAL, clinical attachment loss; MBL: marginal bone loss; PD: pocket depth; PI: plaque index; SD, standard deviations.

CAL results were reported in four of the included studies (BinShabaiba et al., 2019; Javed et al., 2017; Mokeem et al., 2018; Vohra et al., 2020). With exemption to one study which showed no difference between the two groups, all other results for CAL were increased in vaping groups (Vohra et al., 2020). This indicated that subjects who vaped consistently showed more loss of clinical attachment compared to non‐smokers. Differences in probing depth (PD) results were reported in three of the included studies (BinShabaiba et al., 2019; Mokeem et al., 2018; Vohra et al., 2020). Two of these studies showed increased PD in vaping groups while one study showed no difference (BinShabaiba et al., 2019; Mokeem et al., 2018; Vohra et al., 2020). MBL results are divided into mesial and distal measurements, they were reported in all studies with one exception (Alqahtani et al., 2019).

Overall results except for two studies showed increased values in the vaping groups (Javed et al., 2017; Vohra et al., 2020). Interestingly, one study reported mild changes in all periodontal parameters related to vaping (Vohra et al., 2020). This was the only study that was found to show this phenomenon. Most of the studies, as it can be seen from Figures 2, 3, 4, 5, 6, 7, reflected a different trend that alluded to the negative effect of vaping on periodontal tissues.

**FIGURE 8:**
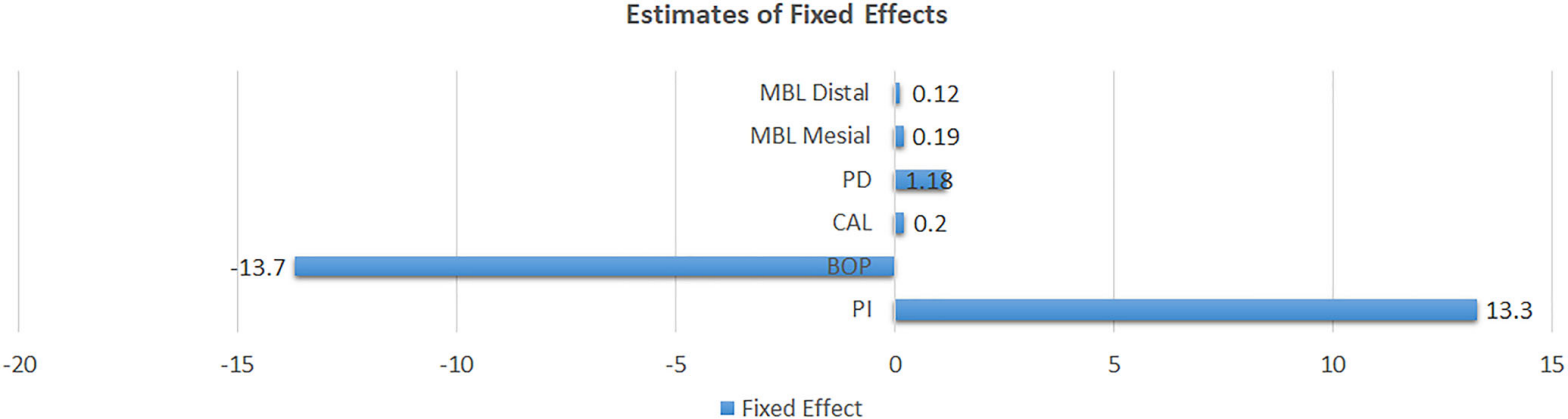
Fixed effect regression model weighted by standard deviations calculated with IBM SPSS. Summary of effects of vaping in each periodontal parameter, where 0 and the bars are a representation of the combined values from Figures 2, 3, 4, 5, 6, 7 after weighting. In this figure, 0 is the control group and the bars are the differences between control groups and vaping groups

Statistical analysis was made with a linear mixed model, and the estimate of fixed effects is shown in Figure 8. The values reported in Figure 8 reflect the combination of all results from individual studies reported in Figures 2, 3, 4, 5, 6, 7, generating one value to represent the effect vaping is causing in each periodontal parameter. Estimate of fixed effect of vapers compared to controls for BOP was 13.73% (*p* < .0001) less, for PI was 13.32% (*p* < .015) more, for CAL was 0.2 mm more (*p* < .5), for PD in % greater than 4 mm was 3.26% more (*p* < .2), PD in mm was 1.18 mm more (*p* < .03) for MBL mesial was 0.19 mm more (*p* < .4) and MBL distal was 0.12 mm more (*p* < .7).

## 4 DISCUSSION

This was the first review conducted to assess the impact of vaping on periodontitis by investigating the changes in periodontal parameters in vape users compared to control groups. Several recent studies have illustrated the effects of vaping on periodontal parameters and we found it relevant to the dental community to have their results combined and analyzed to better understand what impacts to expect from this trend.

Previous studies have compared the effects of vaping to smoking on periodontal parameters, using three group models with cigarette smokers, vape users, and non‐smokers, and found that vape users have results closer to non‐smokers than smokers do (Javed et al., 2017; Subhi et al., 2019). This goes following the results we are seeing in this review, wherewith combined results, vape users did not have exacerbated differences from non‐smokers.

However, there are potentially harmful effects of vaping in general health that should be considered. Besides the previously mentioned metals present in the aerosol, blast injuries, DNA damage, and overproduction of ROS (Kite et al., 2016; Lerner et al., 2016; Shaito et al., 2017), vaping have been linked to cases of severe pulmonary disease in the US (Hammond, 2019).

For the statistical analysis of the extracted results, we assigned weights to each study inversely proportional to their standard deviation. Two of the included studies were assigned the highest weights (Javed et al., 2017; Mokeem et al., 2018). These studies assess CAL, which would be the measurements of choice to detect periodontitis as suggested by the latest classification (Tonetti et al., 2018). The results from these two studies show the most reliable data representing the effects of vaping on periodontal disease in a generalizable context.

The comparison of CAL between the two groups did not show a significant *p* value in the estimate of fixed effects due to the limitation in the number of available results. Whereas, PD, PI, and BOP had significant *p* values. Changes in the PD and PI point to the deleterious effect of vaping on periodontal tissues. Vaping groups present lower BOP when compared to controls. Differences in BOP may be attributed to the presence of nicotine in e‐cigarettes. Nicotine is known to be a vasoconstrictor, which would lead to reduced natural blood flow to the gums and could result in tissue ischemia and impaired healing properties (Silverstein, 1992). A reduction is BOP is a rather negative effect than positive, as gingival bleeding is a symptom that could alarm patients about the need for professional treatment. Without bleeding, the first clinical symptom the patient can perceive is tooth mobility, in a more advanced stage of periodontal disease.

PD results showed deeper pocket depths in vape users compared to control. A deeper pocket site raises a flag for a possible region of inflammation with further tissue destruction. The results from the statistical analysis of this review suggest that vaping might mediate the host's immune response leading to further tissue destruction. Given the popularity vaping has been gaining over recent years, it is important to bring attention to different side effects associated with its use. The papers included in this review were the first ones to analyze the effects of vaping on periodontitis and yield clinical measurement results. However, a few considerations should be taken when interpreting these results.

Firstly, vaping is a relatively new activity, and the duration of the activity until the investigation point may be too short to express all its effects. Also, the selected studies for data extraction were all case–control studies with no follow‐up (Al‐Aali et al., 2018; AlQahtani et al., 2018, 2019; ArRejaie et al., 2019; BinShabaiba et al., 2019; Javed et al., 2017; Mokeem et al., 2018; Vohra et al., 2020). It would be more applicable to have a longitudinal approach to investigate how vaping influences the periodontal status of the users over the years.

Lastly, there was a limited number of available studies, only eight clinical studies investigating vaping effects on periodontal status were eligible. From these, four were at moderate risk of bias (Al‐Aali et al., 2018; AlQahtani et al., 2018; ArRejaie et al., 2019; Vohra et al., 2020). Furthermore, all eight extracted results from a homogeneous population, all groups consist exclusively of males from Saudi Arabia. It must also be noted that in this region, shisha smoking is a very popular activity, and could have influenced these studies results. More studies from different regions are needed to better understand the impacts of vaping on periodontitis.

## 5 CONCLUSION

The effects reported in this review are relatively non‐significant in a clinical context, however, as mentioned earlier, these results should be interpreted with caution, as four of the eight included studies fell into the moderate risk of bias. Although there is not enough evidence to fully characterize the impacts of vaping on periodontitis, the available results point to an unhealthy impact of vaping in the disease, which calls for further clinical studies to assess the effects of vaping on periodontitis longitudinally.

## Data Availability

Data sharing is not applicable to this article as no new data were created or analyzed in this study.
